# A knowledge, attitude, and behaviors survey about healthy living among international students of Erciyes University: a reflection of their respective countries

**DOI:** 10.1186/1753-6561-9-S7-A19

**Published:** 2015-10-27

**Authors:** Munira Omar Salim, Asuman Golgeli, Su Sandar Saing

**Affiliations:** 1 Erciyes University, Faculty of Medicine, Kayseri, Turkey

## Background

Recently Turkey has witnessed an influx of foreign immigrants. This can have an effect in the outcome of health care. We aimed to investigate the knowledge, attitude and behaviors about health among foreign students that have come to Turkey from different countries for collegiate education in order to show at what level these factors play a role in shaping health [1,2].

## Methods

In this survey, 154 international students, 44 girls 108 boys, from 44 different countries studying in 11 different faculties in Erciyes University whose mean age is 21,57±2,88 were evaluated by a questionnaire consisting of five scoops; students use of alcohol and cigarette, knowledge of the use of alcohol and cigarette, eating habits, belief of and attitudes pertaining to healthy living in their countries [3]. Quantitative data obtained was subjected to frequency analysis using SPSS 15.0 and where necessary assessed with cross-analysis.

## Results

Irrespective of country and faculty 70,86% were aware of the negative effects of alcohol and smoking on health.54,30% of the society is engaged in exercises whereas 29,80% agreed that exercising is not practised. 46% are aware of the dangers of obesity whereas 28.66% view it as a sign of wealth. On the importance of breastfeeding, 75% agreed that breastfeeding is widely practised. 30% agree that in their country some people resist immunization due to religous beliefs. 77% agreed to rituals being widely practised for treatment of diseases. 53,70% agreed that evil eye and witchcraft can be the cause of some diseases. 23% agreed to female genital mutilation (FGM) being practised. 35% agreed that there is stigmatisation of diseases like AIDS, leprosy. 61,58% agreed to the direct buying of drugs from chemists without doctor consultation. 68,4% agreed to the use of herbal treatment. 64,5% agreed to the use of family planning methods.

## Conclusions

Despite translocation individuals tend to hold unto their beliefs, attitudes and practises as they try to acculturate [4]. As physicians we have the propensity to practise medicine from a medicocentric point of view. Considering our ever diversifying society it is necessary to incorporate the customs and values of the patients in our care for more favourable outcomes.
